# Visual Information in Basketball Jump-Shots: Differences between Youth and Adult Athletes

**DOI:** 10.5114/jhk/163447

**Published:** 2023-07-06

**Authors:** Marques Rui, Martins Fernando, Gomes Ricardo, Diogo V. Martinho, Mendes Rui, Sarah A. Moore, Manuel J. Coelho-e-Silva, Dias Gonçalo

**Affiliations:** 1 University of Coimbra, FCDEF, Coimbra, Portugal.; 2 Instituto de Telecomunicações, Delegação da Covilhã, Covilhã, Portugal.; 3 Instituto Politécnico de Coimbra, Escola Superior de Educação de Coimbra, Coimbra, Portugal.; 4 Laboratório ROBOCORP, IIA, Instituto Politécnico de Coimbra, Coimbra, Portugal.; 5 University of Coimbra, CIDAF (uid/04213/2020), Coimbra, Portugal.; 6 Faculty of Health, School of Health and Human Performance, Dalhousie University, Halifax, Canada.

**Keywords:** gaze behavior, motor performance, shooting accuracy, eye tracking, vision

## Abstract

Basketball shooting is a complex skill that requires visual routines and trained players typically evidence a specific oculomotor pattern. This study aimed to examine visual patterns in male novice youth and professional adult players while performing a jump shot. The sample included 20 basketball players grouped as under-16 youth (n = 10) and professional adult (n = 10) players. Each participant completed 50 shots at two distances (long range: 6.80 m; middle range: 4.23 m). Eye tracking glasses were used to obtain quiet eye (QE), the number of fixations, total fixation duration, duration of first and last fixation. An independent t-test was used to assess differences between groups. Shooting accuracy given by % of efficacy indicated that under-16 players attained poorer scores at both distances: long (t = −4.75, p < 0.01) and middle (t = −2.80, p < 0.012) distance. The groups also differed in QE time (long: 600 ms vs. 551 ms; middle: 572 ms vs. 504 ms) and total duration of the fixations (long: 663 ms vs. 606 ms; middle: 663 ms vs. 564 ms) in both long and middle distance shots. Significant differences also occurred in the last fixation (long distance: t = −4.301, p < 0.01; middle distance: t = −3.656, p < 0.01) with professional adult players presenting the value of, on average, 454–458 ms, while youth shooters 363–372 ms. In summary, visual strategy differed between under-16 youth and professional adult basketball players. To support their long-term sport development, it is recommended that youth basketball players focus their attention with longer final fixation before releasing the ball to improve their shot.

## Introduction

Shooting is an important skill in basketball. The jump shot corresponds to a dynamic motor skill that combines balance, strength, and technique (França et al., 2021; Okazaki et al., 2009; Krolikowska et al., 2023), and depends on contextual characteristics. Optic variables are particularly important in shooting accuracy (de Oliveira et al., 2008; Klosterman et al., 2019). Experienced players adapt their visual strategy to ensure better manipulation of the ball, adjustment of the body, and extending the preparatory movements according to the position and distance from the basket. Eye-head stabilization is crucial in basketball shooting and often differs between adolescent and adult professional players (Ripoll et al., 1986; Vera et al., 2020). The last phase of the shot is of paramount importance regarding visual information in the control of the motor skill (Okazaki et al., 2015; Oudejans et al., 2002). Vickers (1996a, 2017) described quiet eye (QE) as part of the final fixation between the beginning of the fixation and the first observable movement of the shooting hand. Important variables for players to consider include location of the shooter related to the target, as well as the number and duration of the eye fixation (de Oliveira et al., 2008; Harle et al., 2001; Klostermann et al., 2019) and finally QE time (Vickers, 1996a). Recent research has also explored the influence of anxiety (Gonzalez et al., 2017; Vine et al., 2011, 2014) and attentional focus (Giancamilli et al., 2022; Mann et al., 2007) on the visual search of basketball players while shooting (Moeinirad et al., 2020; Vater et al., 2020). All of these factors may differ between youth and professional adult players.

Before performing the basketball free throw, players fixate their gaze on the rim and the efficacy is associated with the duration and frequency of the fixation (Zwierko et al., 2016). However, the literature about the visual pattern of basketball shooting, age-associated variation is still controversial. Steciuk and Zwierko (2015) concluded that visual search had no more than a partial effect and that fixation frequency was unrelated to skill efficacy in basketball shooting. Meanwhile, another study (Oudejans et al., 2012) suggested that jump-shot efficacy was linked to the duration and frequency of fixations at the rim, as mentioned before for free throws. The fact that QE precedes the beginning of the movement, indicates that this last fixation is used to understand the action output (Gonzalez et al., 2017). The previous applies to sports including projectiles (e.g., golf, basketball) and QE was found to explain performance of experienced professionals in a vast range of shooting actions (Bjelica et al., 2020; Fegatelli et al., 2016; Giancamilli et al., 2022; Kanat et al., 2021). For example, a longer QE period seemed to be associated with better performance and skill acquisition (Vickers et al., 2019; Vine et al., 2013). Therefore, QE may be considered an integral part of specialized motor activities (Vine et al., 2014). The QE of experienced professional adult players is significantly longer than that of youth players (Mann et al., 2007). Moreover, experienced adult players tend to fixate objects in advance and for longer periods than less experienced youth players, regardless of task conditions (Mann et al., 2007; Rienhoff et al., 2015). Longer fixations are associated with better motor programming, particularly on standardized motor tasks such as the free throw. Basketball literature lacks in terms of QE studies regardless of the location (Vickers et al., 2019) and orientation of the shooter and, not surprisingly, Klostermann et al. (2018) questioned the applications of studies to game situations.

Van Maarseveen and Oudejans (2018) examined gaze behaviors of youth basketball players with and without opposition to conclude that shooting accuracy was affected by the duration of the last fixations. In parallel, no differences between under-14 and under-16 players were found in shooting performance (Erčulj and Štrumbelj, 2015). Erčulj and Štrumbelj (2015) suggested that the distance to the basket was an important situational variable, and the age of the participant was positively associated to skill efficacy. Research in basketball has not systematically compared youth and adult professional players. To date, there is limited research comparing the visual patterns of youth players with the visual patterns of more experienced professional adult players (Klostermann et al., 2018; Vickers, 2017; Zwierko et al., 2016). The current study examined the visual search patterns among under-16 youth basketball players and professional adult basketball players. In addition, the study was aimed to identify which variables may support training of youth players. It was hypothesized that visual information would be relevant to discriminate between players considering age- and training-related expertise in basketball shooting, and that differences between players contrasting in training experience would be more apparent in long distance shots.

## Methods

### Ethical Requirements

The research protocol was approved by the Ethics Committee of the Institute Polytechnic of Coimbra (83_CEIPC/2021) and the Scientific Committee of the University of Coimbra Faculty of Sport Sciences and Physical Education. All procedures were in accordance with the ethical standards (World Medical Association, 2013). Participants older than 18 years provided informed consent. Youth participants assented to participate and their parents or legal guardians signed informed consent. Before data collection, basketball players were informed that they could withdraw from the study at any time. Data were always collected at the same indoor basketball court and consistently in the mornings during the pre-competitive period(i.e., the first six weeks of the season). Players wore similar clothing and footwear as usual for their training sessions. Players autonomously completed a 5-min running warm-up including stretching.

### Participants

The sample was composed of 20 male players registered in the Portuguese Basketball Federation. Inclusion criteria were: not injured in the previous month, and being registered in the Portuguese Basketball Federation for at least two seasons. Under-16 players were recruited from representative selection of Portuguese Midlands (n = 10, 7 ± 2 years of training experience; stature: 179.4 ± 2.4 cm; playing position: 4 guards, 6 forwards; all right-hand shooters). The subsample of adult participants was composed of players with a professional contract who played in the Portuguese basketball league (n = 10, 18 ± 5 years of training experience; stature: 193.2 ± 9.8 cm; playing position: 4 guards, 6 forwards; all right-hand shooters).

### Design and Procedures

**Figure 1:**
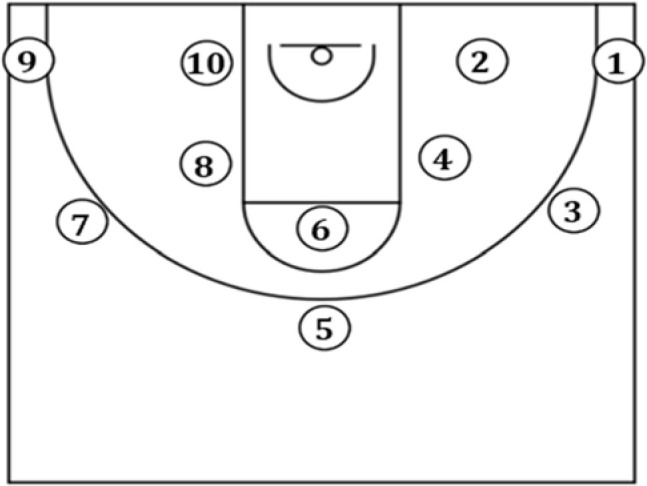
Schematic shot positions (1–10) according to orientation: 0º (positions 1 and 2), 45º (positions 3 and 4), 90º (positions 5 and 6), 135º (positions 7 and 8) and 180º (positions 9 and 10); locations: long distance (positions 1, 3, 5, 7 and 9) and middle distance (positions 2, 4, 6, 8 and 10).

The study was conducted at an indoor training facility fitting official FIBA rules, the particularly sized basketball backboard and the rim placed at a 305-cm height. Each player autonomously completed a brief 5-min warm-up which ended with a shooting routine without eye tracking glasses (ETG). Afterwards, they were fitted with ETG and familiarization was attained by completing several shots until they made five shots under each condition. The official ball selected to complete the motor task was approved by the Portuguese basketball federation (Spalding Co., Bowling Green, USA, Size 7). A 3-point calibration of ETG was made prior to data collection. Participants performed jump shots after receiving the ball (Argiriou et al., 2014) from a skilled observer who had been playing basketball for 10 years. After each rebound, the observer located nearby the basket and completed the chest pass when frontally positioned 3 m away from the shooter to avoid variation due to the dominant and non-dominant pass conditions (Oudejans et al., 2012). These were performed at 4.23 m (middle distance) and 6.80 m (long distance) from the basket, respectively, at five different angles (0º, 45º, 90º, 135º and 180º) as presented in Figure 1. The 5 jump shots were sequentially completed either from positions 1 to 10, or from positions 10 to 1. At each of the 10 positions, five jump shots were performed with a recovery period of 60 s after every 10 trials. Players took the shot immediately after receiving the pass without any attempt to dribble. They were asked to avoid any step prior to the jump shot. Otherwise, the shot was repeated. Thus, after 50 jump shots, the trial was completed.

Eye movements were captured with ETG (SMI ETG 2W; SensoMotoric Instruments, Teltow, Germany). The system recorded eye movements at 60 Hz, and automatically corrected errors with a precision of 0.5º for all distances (iViewETG User Guide, V2.0, of 2013). ETG captured the number and duration of fixations. Software was calibrated to detect a fixation when the gaze dwelled on a location for a minimum of 100 ms. The end of each fixation was determined when the participant produced a saccade, that is a rapid movement between locations in two or more frames (Holmqvist et al., 2011). The last fixation duration corresponded to the final episode immediately before the player dismissed the target for more than 100 ms. Data from blinks were removed (Holmqvist et al., 2011; Di Stasi et al., 2016).

To determine QE times, gaze data were synchronized with the lateral camera, and then manually determined by one trained researcher. QE times were obtained synchronizing ETG with an external camera (Casio Exilim Pro Ex-F1; Shibuya, Tokyo, Japan) with a sampling record of 260 Hz. The camera was positioned laterally, three meters away from the shooting hand. QE time was defined as the time from the beginning of the fixation and the first observable movement of the shooting hand within a minimum of 100 ms (Oudejans et al., 2002). The coding process used ETG proprietary software BeGaze V3.7 (SensoMotoric Instruments, Teltow, Germany). The videos were analysed using a frame-by-frame methodology as described elsewhere (Holmqvist et al., 2011). Adopting the criteria described elsewhere (Marques et al., 2018), the tracking ratio ranged from 93.3 to 99.4% for professional adult players and from 92.4 to 99.6% for under-16 players.

Total fixation duration was determined as the difference between the onset and the end of the fixation. Finally, the number of fixations was given by the sum of all fixations made by a single participant. Shooting accuracy was expressed in percentages as the ratio between the converted shots and the total number of shots (Vickers et al., 2009).

### Statistical Analysis

Descriptive statistics (mean, standard error of mean, 95% confidence intervals and standard deviation) were calculated for the total sample. The Shapiro-Wilk test was used to check the distribution of normality. The Student *t*-test was used for each of the dependent variables derived from ETG in order to compare groups (under-16 youth and professional adult players). The level of significance was set at 5%. For the effect size estimation, Cohen *d*-value was used and interpreted as recommended (Hopkins et al., 2009). All analyses were performed using SPSS version 20.0 (SPSS Inc., IBM Company, NY) and plots were completed using Graphpad Prism (version 5.0 for Windows, GraphPad Software, San Diego, California, www.graphpad.com).

## Results

**Figure 2:**
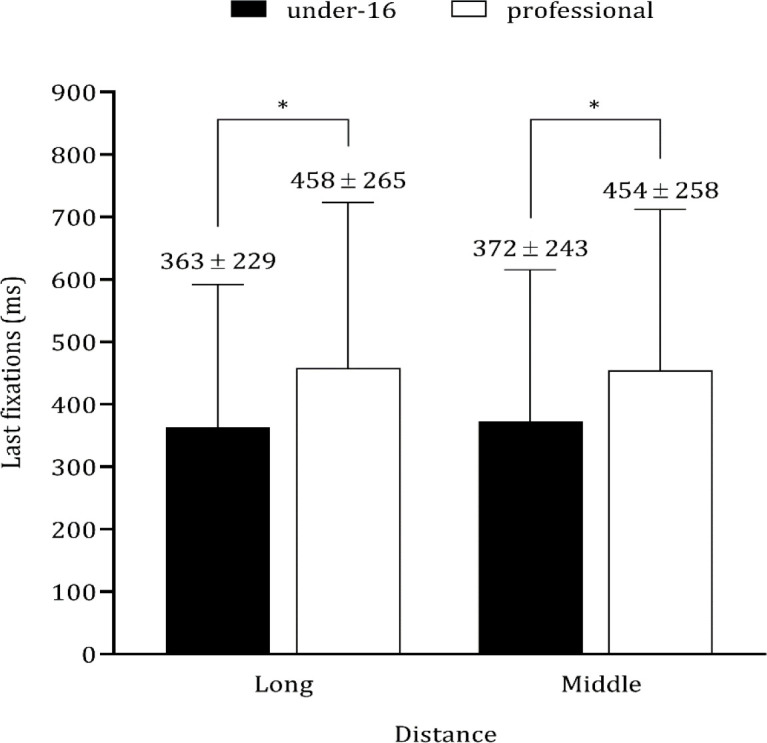
Descriptive statistics (mean ± standard deviation) for quiet eye (QE) time (ms) in first and last fixations in basketball jump shots according to distance from the basket by under-16 and professional players. ^*^ *p* < 0.05

Descriptive statistics for the total sample are summarized in Table 1. Subsequently, Table 2 summarizes comparisons between groups for basketball shooting accuracy. Under-16 youth players compared to professional adult players significantly differed at both distances: middle (t = −2.800, *p* < 0.05), and long range (t = −4.750, *p* < 0.01). As expected, under-16 youth players scored fewer jump shots. QE time also significantly differed between under-16 youth players and professional adult players at both distances (long: t = −2.321, *p* < 0.05; t = −3.258, *p* < 0.01). Additionally, total fixation time differed between under-16 youth players and professional adult players at both distances (long: t = −2.932, *p* < 0.01; and middle: t = −4.767, *p* < 0.01). The number of fixations was significantly greater among youth participants compared to professional players at long distance only (t = 3.197, *p* < 0.01). Finally, the duration of the last fixation significantly differed between the groups at both distances (long: t = −4.301; *p* < 0.01; and middle: t= −3.656; *p* < 0.01, see Figure 2).

**Table 1:** Descriptive statistics for training experience, quiet eye, the total number of fixations and duration of fixations (total, first and last) for the total sample (n = 20).

Variables (Xi)	Unit	Mean			SD	Normality	
		value	SEM	(95%CI)		Shapiro-Wilk value	*p*
Training experience	years	13	1.5	(10 to 16)	6.9	0.910	0.064
Quiet eye	ms	564	22.4	(517 to 610)	100.3	0.948	0.337
Fixations number	#	1.95	0.08	(1.77 to 2.12)	0.37	0.971	0.769
Fixations total	ms	729	40.2	(644 to 813)	179.9	0.944	0.281
Last fixation	ms	407	28	(349 to 466)	124	0.969	0.744

SEM (standard error of the mean); 95%CI (95% confidence interval); SD (standard deviation).

**Table 2:** Descriptive statistics (mean ± standard deviation) for basketball shooting accuracy (in %), QE time (ms), duration (ms) and the number of fixations according to distance from the basket among under-16 (n = 10; 500 shots) and professional players (n = 10; 500 shots); results of *t*-test and effect sizes.

Yi:		X: independent variable (age groups)		*t*-test		Magnitude effect	
Dependent variable (unit)	Distance	Under-16	Professional	*t*-value	*p*	*d*	(qualitative)
Shooting accuracy (%)	Long	33.6 ± 13.6	58.8 ± 9.8	4.750	0.001	2.12	(very large)
	Middle	52.0 ± 10.5	69.2 ± 16.3	2.800	0.012	1.25	(large)
QE time (ms)	Long	551 ± 230	600 ± 249	2.321	0.021	0.215	(small)
	Middle	504 ± 238	572 ± 233	3.258	0.001	0.304	(small)
Fixations: total duration (ms)	Long	606 ± 195	663 ± 243	2.932	0.004	0.273	(small)
	Middle	564 ± 221	663 ± 245	4.767	0.001	0.447	(small)
Fixations (number)	Long	2.11 ± 0.93	1.87±0.74	3.197	0.001	0.301	(small)
	Middle	1.88 ± 0.85	1.99 ± 0.75	0.833	0.405	0.145	(trivial)
Last (ms)	Long	363 ± 229	458 ± 265	4.301	0.001	0.404	(small)
	Middle	372 ± 243	454 ± 258	3.656	0.001	0.345	(small)

## Discussion

The present study examined differences in visual information between under-16 youth basketball players and professional adult basketball players. As expected, the older and more experienced group attained better scores in jump shot efficacy. The study also observed differences between youth and professional adult players on the number and duration of fixations and QE time. In fact, QE time was longer and the same occurred for the total fixation duration among professional players. The last fixation duration was shorter in youth players compared to professional adult participants at both distances. Under-16 youth players had a greater number of fixations, though this was evident in the long, but not the middle distance.

The mechanisms underlying expert performance have aroused much interest. In many sports, skilled perception precedes and determines appropriate action (Kambič et al., 2022). In the current study, it could not be intuitively assumed that best shooters would be endowed with better visual skills per se, but rather their enhanced cognitive knowledge. The preceding enables them to pick up and interpret perceptual information more effectively than less skilled shooters. The complex interactions between perception, cognition, and expertise have been previously tested among youth soccer players aged 13–15 years (Ashford et al., 2021; Vaeyens et al., 2007a). The previous study (Vaetens et al., 2007a) assigned players as successful or less successful on the basis of their performance on a tactical skill test in the laboratory using soccer-specific film simulations and eye movement registration techniques. It was concluded that when compared to less successful counterparts, successful decision-makers used more goal-oriented visual search strategies and were characterized by faster decision, greater response accuracy with more time fixating the player in ball possession. In addition, playing experience, the skill level, and the constraints of the task (including the number of players and the relative proportion of offensive and defensive players) were considered determinants of visual search in the dynamics of offensive team simulations (Ashford et al., 2021; Vaeyens et al., 2007b).

The current study compared the visual search while performing jump shots among under- 16 youth and professional adult basketball players. Dependent variables included variation in the distance of the shooter from the basket. Briefly, professional players evidenced fewer fixations and longer time of the final fixations compared with youth players. The results need to be confirmed while catching the ball after dribbling with and without opponents. The effect of the action preceding the jump shot was investigated among female basketball players (Erčulj et al., 2020; Oudejans et al., 2012), and it was concluded that shooting was more complex after dribbling which often occurred with an opposition (1 x 1 situation). Previous research of female basketball players (Vickers, 1996a, 1996b, 2017) classified each participant as experts (n = 8) and near experts (n = 8) to examine the free throw condition. Experts evidenced longer fixation on the target. Meantime, the interrelationship among control of gaze during sport-specific tasks and the perceptual and cognitive processes was also studied (Vickers, 2011; Vickers et al., 2019). QE was underlined as a characteristic of a higher level of performance with the final fixation or tracking gaze occurring prior to the final movement. The basketball literature also examined three-point shooting (Vickers, 2009) with the experimental set up covering different QE periods corresponding to the phases of the motor task: QE catch, QE arm preparation, QE arm flexion, QE arm extension, and QE ball release. The sample was composed of 12 elite female basketball players who completed 40 three-point jump shots after receiving a pass under different conditions: uncontested (without an opponent) versus contested and, as expected, results confirmed lower accuracy levels under the contested condition, in part, because the defender affected the duration of QE periods and release time. In this sense, coaches need to train young basketball players in the refinement of the sequence: reception, visual search option, and shooting.

Previous research on the topic using this technology investigated the effects of the presence of the opponent on shooting performance in 12 male elite basketball players aged 22 years (Vickers et al., 2019). In fact, sample size seems a limitation in many available studies focused on visual strategies in sport. Although caution is needed in the generalization of the findings, they appear to be of substantial relevance in determining the QE duration of professional adult basketball players. Visual strategies exhibited by amateur versus professional players or youth versus adult participants in competitive basketball can be interpreted as training materials by selecting pedagogical sequences. Future comparisons between youth and adult professional basketball players with larger samples are needed. In addition, younger and older groups such as under-14 and under-18 would allow information by age groups covering all competitive categories. Moreover, groups of contrasting training experience within each age group would also be interesting to test. Such studies should consider years of formal training and also accumulated experience in recreational basketball. Youth players in the current study were not exposed to specialization in terms of the playing position and no player was classified as a center. It could be hypothesized that guards, forwards and centers may develop different routines in shooting skills: after dribbling or after receiving a pass, from the front or the baseline, after a screen. It is plausible that each condition would require distinct visual information.

QE provides crucial information regarding the utility of gaze behavior for the decision-making process and is, consensually, considered a key factor in the perception-action complex (Causer et al., 2011; Vickers, 2007). Despite the fact that in the current study, under-16 basketball players corresponded to the regional elite level, another topic for future studies would be to examine the best shooters at different competitive ages (under-14, under-16, under-18). Relevant information would be added to previous studies (Fegatelli et al., 2016; Mann et al., 2007; Rienhoff et al., 2015; Vickers, 1996a, 1996b, 2009) by comparing expert versus non-expert players performing a sport-specific task. It should be noted that not all basketball players playing in the perimeter are exceptional shooters. The topic should be viewed as crucial to attain the professional level and will support coaches to train adolescent players in visual strategies for success. A long fixation on a specific location prior to the movement allows to capture specific visual information crucial for the organization, programming, initiation and control of the movement (Klostermann et al., 2013; Vickers et al., 2019; Wilson and Vine, 2018). An external focus refers to attention to the environment. Hence, in soccer, attentional focus strategies were demonstrated to influence superior performance, particularly in goalkeepers who deal with complex constraints imposed by the opponents (Marques et al., 2018). On the other hand, when instructed with an internal focus, self-reflection contributes to gain a better knowledge about motor abilities and performance (Kupper et al., 2020; Wulf and Lewthwaite, 2009). Coaches need to recognize how youth players respond in the context characterized by different levels of incertitude and complexity (Wulf, 2013).

## Conclusions

The current study identified a specific visual search pattern among professional adult basketball players when compared to youth participants. As a practical implication, basketball coaches need to include visual search strategies while training youth players, particularly the fixation on the target until the end of the motor action. Visual strategies should be progressively refined by introducing opponents with multiple combination of shooting distances and directions after dibbling or after receiving the ball.
